# Identification of a reticulocyte-specific binding domain of *Plasmodium vivax* reticulocyte-binding protein 1 that is homologous to the PfRh4 erythrocyte-binding domain

**DOI:** 10.1038/srep26993

**Published:** 2016-05-31

**Authors:** Jin-Hee Han, Seong-Kyun Lee, Bo Wang, Fauzi Muh, Myat Htut Nyunt, Sunghun Na, Kwon-Soo Ha, Seok-Ho Hong, Won Sun Park, Jetsumon Sattabongkot, Takafumi Tsuboi, Eun-Taek Han

**Affiliations:** 1 Department of Medical Environmental Biology and Tropical Medicine, School of Medicine, Kangwon National University, Chuncheon, Gangwon-do, Republic of Korea; 2 Department of Clinical Laboratory, The First Affiliated Hospital of Anhui Medical University, Hefei, Anhui, People’s Republic of China; 3 Department of Medical Research, Yangon, Myanmar; 4 Department of Obstetrics and Gynecology, School of Medicine, Kangwon National University, Chuncheon, Gangwon-do, Republic of Korea; 5 Department of Molecular and Cellular Biochemistry, School of Medicine, Kangwon National University, Chuncheon, Gangwon-do, Republic of Korea; 6 Department of Internal Medicine, School of Medicine, Kangwon National University, Chuncheon, Gangwon-do, Republic of Korea; 7 Department of Physiology, School of Medicine, Kangwon National University, Chuncheon, Gangwon-do, Republic of Korea; 8 Mahidol Vivax Research Unit, Faculty of Tropical Medicine, Mahidol University, Bangkok, Thailand; 9 Division of Malaria Research, Proteo-Science Center, Ehime University, Matsuyama, Ehime, Japan

## Abstract

The *Plasmodium vivax* reticulocyte-binding protein (RBP) family was identified based on the annotation of adhesive ligands in the *P. vivax* genome. Reticulocyte-specific interactions with the PvRBPs (PvRBP1 and PvRBP2) were previously reported. *Plasmodium falciparum* reticulocyte-binding protein homologue 4 (PfRh4, a homologue of PvRBP1) was observed to possess erythrocyte-binding activity via complement receptor 1 on the erythrocyte surface. However, the reticulocyte-binding mechanisms of *P. vivax* are unclear because of the large molecular mass of PvRBP1 (>326 kDa) and the difficulty associated with *in vitro* cultivation. In the present study, 34 kDa of PvRBP1a (PlasmoDB ID: PVX_098585) and 32 kDa of PvRBP1b (PVX_098582) were selected from a 30 kDa fragment of PfRh4 for reticulocyte-specific binding activity analysis. Both PvRBP1a and PvRBP1b were found to be localized at the microneme in the mature schizont-stage parasites. Naturally acquired immune responses against PvRBP1a-34 and PvRBP1b-32 were observed lower than PvDBP-RII. The reticulocyte-specific binding activities of PvRBP1a-34 and PvRBP1b-32 were significantly higher than normocyte binding activity and were significantly reduced by chymotrypsin treatment. PvRBP1a and 1b, bind to reticulocytes and that this suggests that these ligands may have an important role in *P. vivax* merozoite invasion.

*Plasmodium* spp. cause public health problems worldwide, especially in tropical and subtropical nations. *Plasmodium vivax* has been reported to be the most widespread cause of malaria worldwide, with a harmful influence on an estimated 124–283 million people^1^. *P. vivax* is neglected compared to *Plasmodium falciparum* because it is associated with relatively low mortality; however, it is the most broadly and continuously spread species globally^2^,^3^.

The investigation of specific interactions between parasite ligands and red blood cell (RBC) receptors is important to elucidate the complicated invasion mechanisms involved in multiple processes during the asexual erythrocytic stage of the malaria parasite^4^. *P. vivax* preferentially interacts with reticulocytes (young RBCs) during the repetitive invasion process, whereas *P. falciparum* is able to invade all stages of RBCs in circulation. Specific interactions between the *P. vivax* ligand, Duffy binding protein (DBP) and RBC receptor Duffy antigen/receptor for chemokines (DARC) were reported to be essential for invasion^5^,^6^. However, recently, Duffy-negative Malagasy clinical cases involving *P. vivax* infection have been reported, indicating that *P. vivax* may have an alternative invasion pathway^7^. One possible alternative pathway is mediated by the reticulocyte-binding protein (RBP) family. PvRBP1 and PvRBP2 were identified as essential parasite ligands from this family that selectively bind reticulocytes^8^,^9^. Whole-genome annotations of PvRBP1 (PvRBP1a, PvRBP1b and PvRBP1 partial-1) and PvRBP2 (PvRBP2a, PvRBP2b, PvRBP2c, PvRBP2 partial-1, and PvRBP2 partial-2) have been completed and used to reveal promising vaccine candidates^10^,^11^. Analysis of the *pvrbp1a* and *pvrbp1b* (PVX_098585 and PVX_098582) amino acid sequence structures revealed that PvRBP1 contained two exons; the first exon encoded a signal peptide, and the second exon encoded a hydrophobic sequence (transmembrane domain) at the C-terminal region and an arginyl-glycyl-aspartic acid (RGD) motif 8. PvRBP1a and PvRBP1b are highly transcribed during the parasite schizont stage^10^,^11^,^12^, suggesting that these proteins play important roles in reticulocyte invasion by blood stage parasites. However, the involved binding motif and whether the PvRBP proteins interact with reticulocytes have remained largely unknown. One study demonstrated robust invasion assay which has allowed testing molecules in invasion of 2C3 as the monoclonal antibody of DARC for blocked PvDBP interaction by short term *P. vivax* invasion process^13^. An invasion mechanism study of *P. vivax* faces a considerable hurdle owing to the inability to continuously culture the parasites *in vitro*^14^,^15^. To overcome these problems, we consider evidence from homologous protein families in other *Plasmodium* spp.

Reticulocyte binding-like (RBL) homologues have been found among human-, simian- and rodent-infecting *Plasmodium* spp.^9^,^16^,^17^. This highly consistent function from adhesive protein family members was based on the binding activity toward erythrocytes by *P. falciparum* and may provide clues that the PvRBP1s also play essential roles in parasite invasion through ligand-receptor interactions^16^,^18^,^19^. The erythrocyte-binding domain of reticulocyte-binding protein homologue 4 (PfRh4) from *P. falciparum* was identified as a homologous region to the PvRBP1 amino acid sequence. This domain showed erythrocyte-binding activity and was specifically inhibited by antibodies^16^. PfRh4 interacted with the CCP1-3 site recognized by the complement receptor type 1 (CR1) on the erythrocyte surface via a sialic acid-independent invasion pathway. Several *P. falciparum* strains primarily use sialic acid-independent pathways for RBC invasion^20^,^21^,^22^. In PfRh5, erythrocyte-binding activity via basigin was demonstrated from the PfRh4-binding homologue site^23^,^24^. Recently, analysis of the PvRBP2a crystal structure showed structural conservation of the PfRh5 scaffold shape^25^,^26^. Interestingly, all PvRBP family members (PvRBP2a, PvRBP2b, PvRBP2c, PvRBP2 partial-1, PvRBP2 partial-2, PvRBP1a and PvRBP1b) share this protein structure at the N-terminal region; of these PvRBPs, PfRh4, PfRh5 and PvRBP2a showed erythrocyte-binding activities^26^. All PfRh family members (PfRh1, PfRh2a, PfRh2b, PfRh3, PfRh4, and PfRh5) with homologous domains revealed erythrocyte binding activities except PfRh3 (pseudogene)^18^,^19^,^23^,^27^. Overall, several studies have provided strong evidence for the involvement of this protein family in erythrocyte or reticulocyte binding. However, the characterization and identification of PvRBP1a and PvRBP1b are insufficient compared with other RBL family members.

In this study, we characterized PvRBP1a and PvRBP1b as PfRh4 erythrocyte-binding domain homologue regions. We demonstrated their exact subcellular localization in blood-stage parasites, their ability to acquire immune responses in malaria patients, and their binding activity with normocytes and reticulocytes under *in vitro* conditions.

## Results

### Schematic structures of PvRBP1a and PvRBP1b

**Figure 1:**
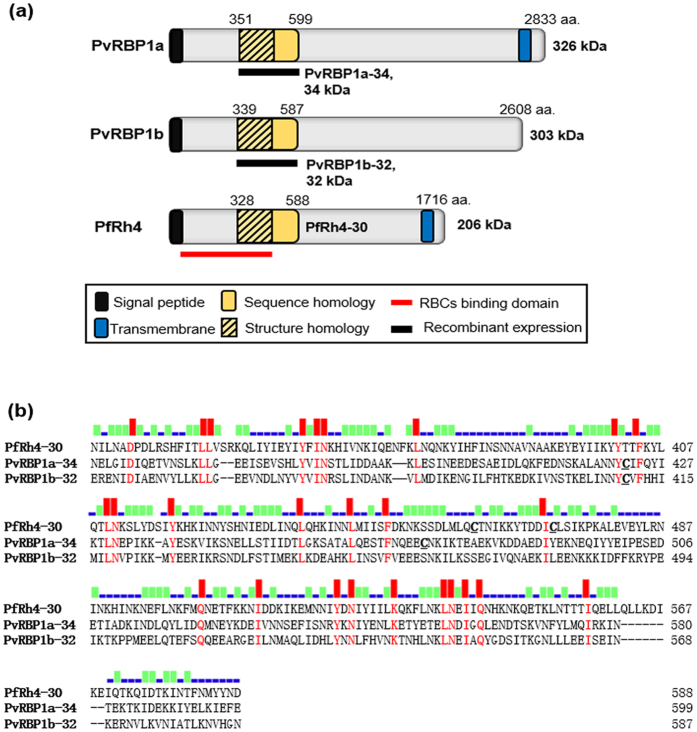
Schematic structure and amino acid sequence alignments of PvRBP1a, PvRBP1b and PfRh4. (**a**) The PvRBP1a-34 (351–599 amino acid [aa.]) and PvRBP1b-32 (339–587 aa.) sequences homologous with PfRh4-30 (328–588 aa.) are represented in a yellow box. The signal peptide (black box), transmembrane domain (blue box), structure homologue (lined box), recombinant protein expression (black bar) and functional erythrocyte-binding domain (red bar) are indicated. (**b**) Clustal alignment of the PvRBP1a, PvRBP1b and PfRh4 homologous sequence domains. The red bar indicates the conserved identical amino acids in the alignment of the three proteins, the green bar indicates identical amino acids in two proteins, and the blue bar indicates diverse amino acids in the three proteins.

The PvRBP1a and PvRBP1b gene sequences encode large-sized proteins (2,833 and 2,608 aa., respectively) with predicted high molecular weights (approximately 326 and 303 kDa, respectively). PvRBP1a encodes a signal sequence (1–22 aa.) and transmembrane domain (2,756–2,774 aa.) and contains 16 cysteine residues. PvRBP1a-34 (351–599 aa.) is a putative functional domain homologue site of PvRBP1a and was defined based on the PfRh4-30 homologue site as an erythrocyte-binding domain. PvRBP1b also encodes a signal sequence (1–23 aa.) and contains 12 cysteine residues. PvRBP1b-32 (339–587 aa.) is a predicted binding domain of PvRBP1b and was selected from the PvRBP1a-34 homologue region (Fig. 1a). The sequence alignment of PfRh4-30 with PvRBP1a-34 and PvRBP1b-32 generated using Clustal W and including the 600 aa. upstream of the N-terminal region showed that PfRh4-30 has 17.2% sequence identity with PvRBP1a-34, PfRh4-30 has 18.4% identity with PvRBP1b-32, and PvRBP1a-34 has 37.5% identity with PvRBP1b-32 (Fig. 1b). This putative binding sequence was predicted to represent the conserved domain in PvRBP1a (354–459 aa.) and PvRBP1b (342–450 aa.) that was present in the PfRh4-30 (330–440 aa.) 3D structure (Supplementary Fig. S1). These conserved structure domains also overlap the binding domain of the PfRh5 (160–506 aa.) monoclonal antibody (PBD template c4u1gA) (Supplementary Fig. S1).

### Expression and purification of recombinant PvRBP1a-34 and PvRBP1b-32

**Figure 2:**
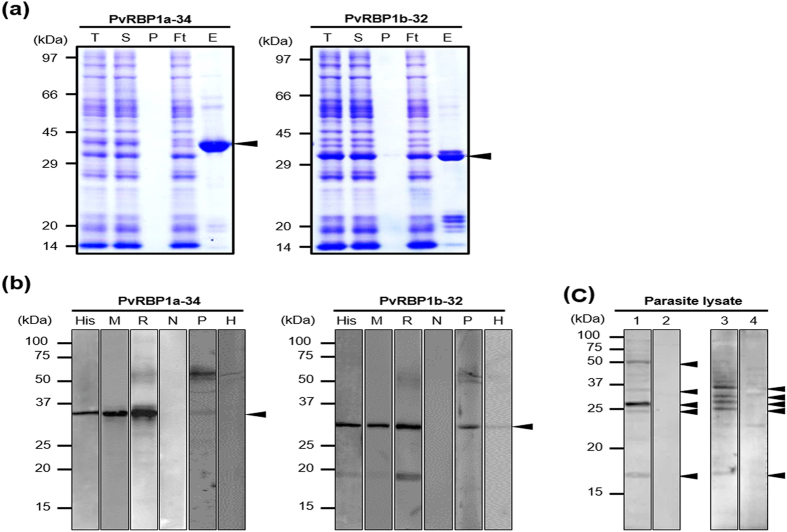
Recombinant PvRBP1a-34 and PvRBP1b-32 expression and western blotting of recombinant proteins and *P. vivax* schizont extracts with specific antibodies. (**a**) Recombinant protein expression and purification. The arrowhead indicates the specific bands for the purified PvRBP1a-34 (34.4 kDa) and PvRBP1b-32 (32.0 kDa) recombinant proteins. T, total translation mix; S, supernatant; P, pellet; Ft, flow-through; E, elution. (**b**) The purified recombinant PvRBP1a-34 and PvRBP1b-32 proteins were probed with the anti-penta-His antibody (His), immune mouse sera (M), immune rabbit sera (R) and preimmune rabbit sera (N) under reducing conditions as well as pooled patient sera from 10 vivax malaria patients (P) and pooled sera from 10 healthy people (H). (**c**) Recognition of the native PvRBP1a and PvRBP1b antigens in the *P. vivax* schizont parasite lysate with rabbit antisera raised against the recombinant PvRBP1a-34 and PvRBP1b-32 proteins under reducing conditions. (1) Native PvRBP1a with rabbit immune sera; (3) native PvRBP1b with rabbit immune sera; (2) and (4) represent normal RBC extracts with PvRBP1a and PvRBP1b rabbit immune sera, respectively. The arrowhead represents the target bands and their processed fragments.

The recombinant PvRBP1a-34 and PvRBP1b-32 proteins were expressed in the wheat germ cell-free system and purified with a Ni-NTA affinity column (Qiagen, Hilden, Germany). The PvRBP1a-34 and PvRBP1b-32 fragments had expected molecular weights of approximately 34.4 kDa and 32.0 kDa, respectively, and were successfully expressed in their soluble forms (Fig. 2a). The recombinant protein purification was confirmed by western blotting with an anti-His antibody (Fig. 2b, Lane His). These recombinant proteins were used to immunize mice and rabbits to produce polyclonal antibodies. The immune sera raised against the recombinant proteins reacted with the PvRBP1a-34 and PvRBP1b-32 proteins (Fig. 2b, Lane M and R). Similarly, mixed serum samples from 10 *P. vivax*-infected patients specifically recognized recombinant PvRBP1a-34 and PvRBP1b-32. In contrast, neither pre-immune animal sera nor sera from healthy humans reacted with the recombinant antigens (Fig. 2b, Lane N). Immune sera raised against PvRBP1a-34 and PvRBP1b-32 specifically recognized their native antigens from *P. vivax* schizont-enriched lysates (Fig. 2c).

### Humoral immune response against PvRBP1a-34 and PvRBP1b-32

**Figure 3:**
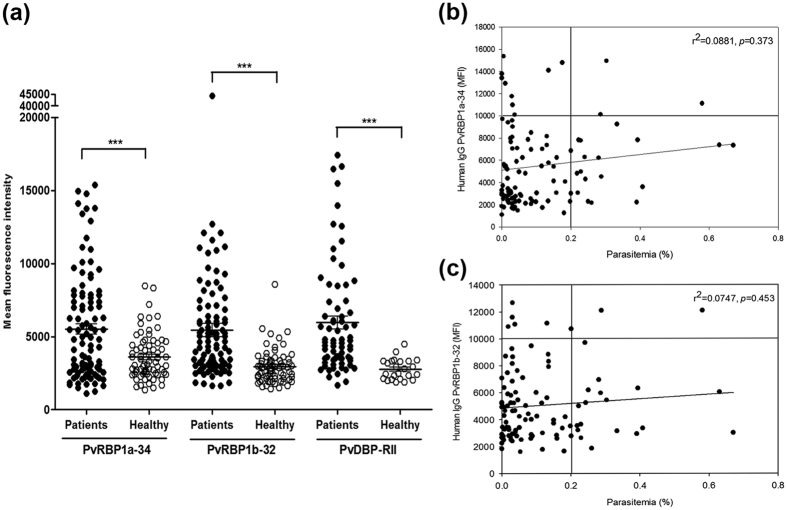
Antigenicity and correlation of parasitemia with PvRBP1a-34 and PvRBP1b-32. (**a**) Total IgG prevalence of each domain with the vivax malaria patient sera and healthy individual samples. The bar indicates the mean ± standard deviation of the mean fluorescence intensity (MFI). The *p* values were calculated using Student’s *t*-test. (**b**) The correlation between PvRBP1a-34 and (**c**) PvRBP1b-32 with parasitemia was evaluated using Spearman’s correlation test. An MFI >10,000 was considered high intensity and >0.2 was considered high parasitemia.

The antigenicity of the PvRBP1a and PvRBP1b fragments was evaluated by protein array. For this experiment, serum samples were obtained from 104 vivax malaria patients and 72 healthy individuals in the Republic of Korea (ROK). The total IgG prevalences of PvRBP1a-34 and PvRBP1b-32 were 33.7% (mean fluorescence intensity; mean ± 2 standard deviation [S.D.], 3,593 ± 3,112) and 39.4% (mean ± 2 S.D.; 2,940 ± 2,296) sensitivity, respectively, with 95.8% specificity. Both proteins had high specificity, and significant differences were observed in the total IgG prevalence between the vivax patients and healthy individuals (*p* < 0.0001) (Table 1; Fig. 3a). PvDBP-RII was selected for the antigenicity analysis for comparison. For the antigenicity screening, sera from 72 vivax patients were randomly chosen from the 104 samples. These data re-confirmed the high IgG prevalence against PvDBP-RII with 56.9% sensitivity and 95.8% specificity (Table 1; Fig. 3a). There was no significant correlation between the parasitemia and the humoral immune response to PvRBP1a-34 (*r*^2^ = 0.0881, *p* = 0.373) and PvRBP1b-32 (*r*^2^ = 0.0747, *p* = 0.453) (Fig. 3b,c).

### Subcellular localization of PvRBP1a and PvRBP1b

**Figure 4:**
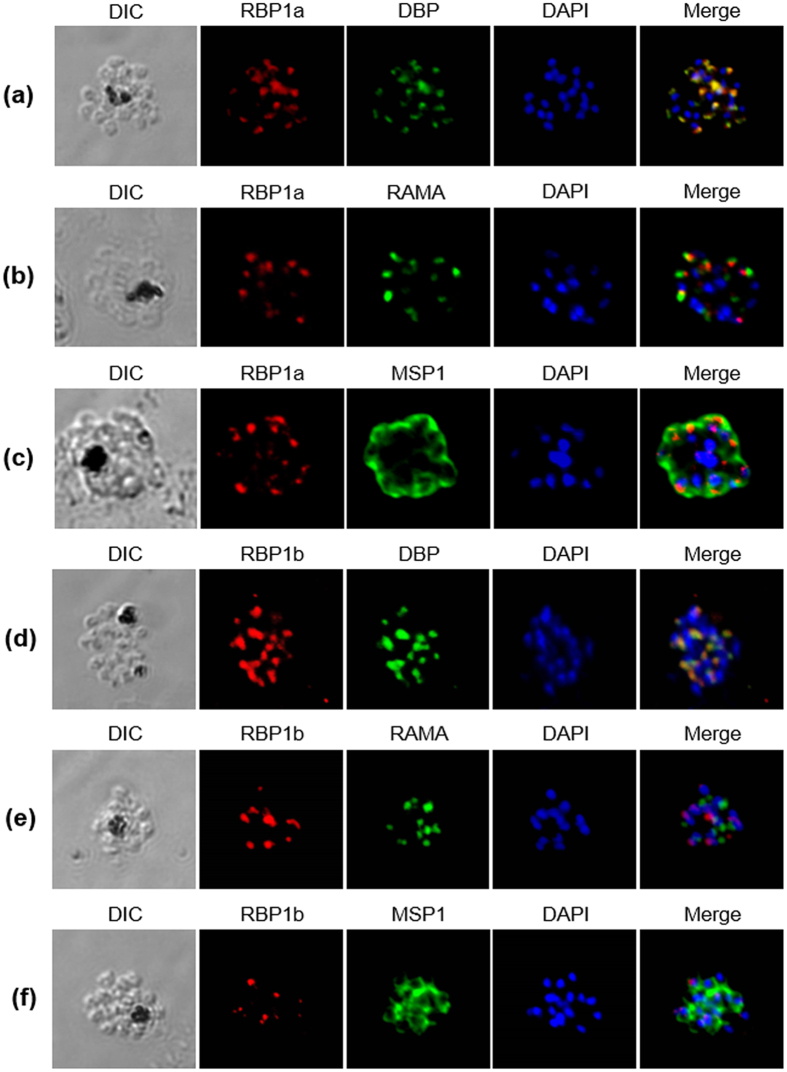
Localization of PvRBP1a and PvRBP1b. (**a–f**) Subcellular localization of the PvRBP1a and PvRBP1b proteins in asexual blood-stage *P. vivax* parasites. PvRBP1a or PvRBP1b rabbit immune sera (red color) were dual labeled with mouse immune sera against PvMSP1-19 (merozoite surface), PvRAMA (rhoptry) and PvDBP-RII (microneme) (green). The nuclei are visualized with DAPI (blue).

The subcellular localization of PvRBP1a and PvRBP1b in the merozoite parasites was observed using the anti-PvRBP1a-34 and anti–PvRBP1b-32 antibodies by confocal microscopic examination. Native PvRBP1a colocalized on the microneme of the merozoite together with the PvDBP microneme protein marker (Fig. 4a). The merozoite surface and rhoptry protein markers (PvMSP1 and PvRAMA, respectively) did not merge together (Fig. 4b,c). PvRBP1b also clearly colocalized with PvDBP (Fig. 4d) but not with PvRAMA or PvMSP1 (Fig. 4e,f).

### Reticulocyte-binding activity of PvRBP1a-34 and PvRBP1b-32

**Figure 5:**
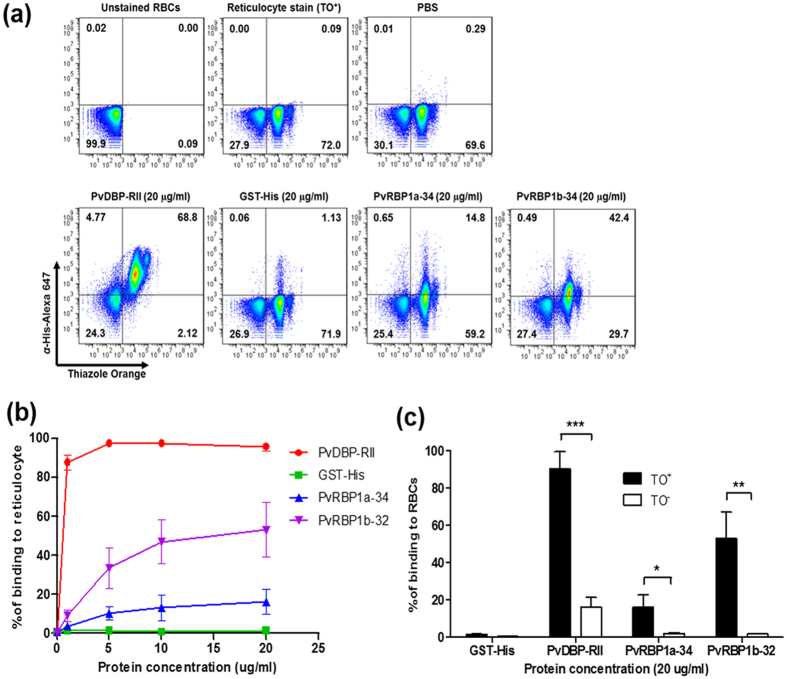
Reticulocyte-binding activity of recombinant PvRBP1a-34 and PvRBP1b-32 in the FACS-based assay. (**a**) Dot plot indicates the unstained RBCs as the gating control (upper left), the enriched reticulocytes (thiazole orange, TO^+^) (upper center) and the binding control containing no protein (phosphate-buffered line, PBS) in the added fractions (upper right). Recombinant proteins (20 μg/ml) added to the test group are shown in the lower image. (**b**) Reticulocyte-binding saturation assay showing the total binding percentage of reticulocytes with serial concentrations of the proteins. The PvDBP-RII and GST-His proteins were used as positive and negative controls, respectively. Data are shown as the mean ± standard deviation (S.D.) of at least three independent experiments. (**c**) Comparison between TO^+^ and TO^−^ (reticulocytes and normocytes, respectively) binding activity with 20 μg/ml of each protein. Data are shown as the mean ± S.D. of at least three independent experiments.

The reticulocyte-binding activity evaluation of PvRBP1a-34 and PvRBP1b-32 was performed using a fluorescence-activated cell sorter (FACS)-based binding assay. The reticulocytes were enriched from cord blood, and a mean concentration of 73.3% (95% confidence interval, 61.3–85.3%) was achieved (Fig. 5a). PvDBP-RII and the His-tagged GST protein were used as the positive and negative controls, respectively, for the binding activity evaluation. PvDBP-RII dramatically bound to reticulocytes at the 1 μg/ml protein concentration and was saturated at the approximately 5 μg/ml concentration (Fig. 5b). The PvRBP1a-34 and PvRBP1b-32 binding activities on reticulocytes were shown to increase in a concentration-dependent manner, and both proteins were saturated at the 20 μg/ml concentration (Fig. 5b). PvDBP-RII has well-known binding activities with normocytes and reticulocytes via DARCs. PvDBP-RII binding was detected at the 20 μg/ml concentration with a mean binding of 16.2% ± 5.2% with normocytes and 90.5% ± 9.0% with reticulocytes (*p* < 0.0002), which represented a 5.6-fold increase in the reticulocytes (Fig. 5c). The PvRBP1a-34 and PvRBP1b-32 proteins specifically bound to reticulocytes (16.2% ± 6.4% and 53.1% ± 14.0%, respectively) and were significantly higher reticulocyte binding activity than normocyte-binding activity (*p* < 0.0178 and *p* < 0.0032, respectively) (Fig. 5c). The normocyte-binding activity of the two PvRBP1 proteins was similar to that of GST-His and was below the cut-off percentage that indicated no binding activity with normocytes. The GST-His protein was included as the negative control and was used as the binding cut-off value for the normocyte-binding activity analysis. The GST control was shown to have very low binding activity in the normocyte and reticulocyte populations, respectively.

**Figure 6:**
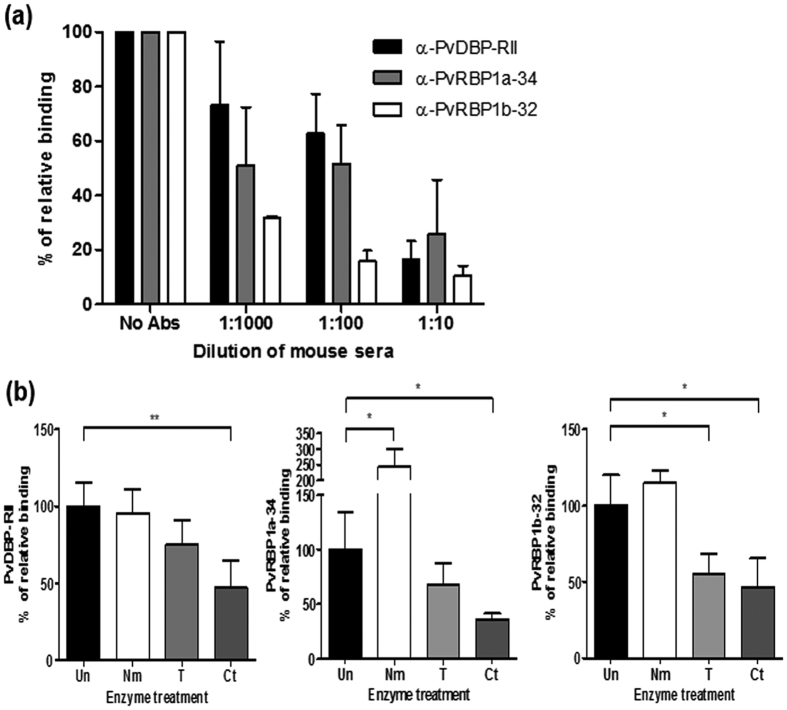
Specificities of PvRBP1a- and PvRBP1b-binding and reticulocyte receptors. (**a**) Antibody inhibition assay. Serial dilutions of mouse sera raised to inhibit the protein-binding activity. Data are shown as the mean ± S.D. of at least three independent experiments. (**b**) A flow cytometry-based RBC-binding assay was performed with untreated RBCs (Un) and neuraminidase- (Nm), trypsin- (T), and chymotrypsin (Ct)-treated RBCs. Significant differences are shown as single asterisks, *p* < 0.05; and double asterisks, *p* < 0.01. Data are shown as the mean ± S.D. of at least three independent experiments.

The specificity of the reticulocyte-binding activity was confirmed by the binding inhibition assay. The antibodies against PvDBP-RII, PvRBP1a-34 and PvRBP1b-32 were able to inhibit the binding of each protein in a concentration-dependent manner (Fig. 6a). The recombinant proteins bound to reticulocytes treated with neuraminidase, trypsin or chymotrypsin. The relative binding activity of the chymotrypsin-treated reticulocytes with PvDBP-RII was inhibited by more than half compared to PvDBP-RII (percentage of relative binding, mean + SD: 47.5% + 17.1%); this binding activity was significantly different (*p* = 0.004) than the binding activity for normal reticulocytes. Similarly, PvRBP1a-34 (35.9% + 5.6%) was significantly inhibited by chymotrypsin (*p* = 0.033). The binding activity of PvRBP1b-32 was significantly inhibited by trypsin (55.4% + 13.3%, *p* = 0.033) and chymotrypsin (46.7% + 18.9%, *p* = 0.029), similar to the PfRh4-30 erythrocyte-binding domain (Fig. 6b).

## Discussion

*P. vivax* invades host red blood cells via complex mechanisms, such as the recognition and interaction of reticulocytes with merozoites in the blood stream. The most suitable invasion mechanism candidates were PvRBP1 and PvRBP2 in a previous report^8^. These proteins demonstrated concentration-dependent reticulocyte-binding activity and accordingly demonstrated a preference for interactions with reticulocytes rather than normocytes. PvRBP homologues have been found in various human (*P. vivax* and *P. falciparum*), simian (*P. cynomolgi* and *P. knowlesi*) and rodent (*P. berghei* and *P. yoelii*) malarial agents. The first member of this RBL family was identified in a rodent malarial agent (*P. yoelii*), and its function was identified as reticulocyte invasion^28^. The Rhoptry protein with a 235 kDa molecular weight (PY235) identified in *P. yoelii* was involved in host cell selection for invasion^29^,^30^. Subsequently, multigene family paralogs were identified in *P. vivax,* including PvRBP1 and PvRBP2^9^. The complete genome of the *P. vivax* Sal-I strain was sequenced, and three *pvrbp1* genes were found: PvRBP1a (PVX_098585), PvRBP1b (PVX_098582) and PvRBP1 partial (PVX_125738)^11^. *P. falciparum* reticulocyte-binding protein homologues (PfRh family; PfRh1, PfRh2a, PfRh2b, PfRh4, and PfRh5) were also detected in PvRBPs^17^,^31^,^32^,^33^,^34^ and showed erythrocyte-binding activity. PfRh4 contained an erythrocyte-binding domain that was homologous to the PvRBP1 site^16^,^21^,^22^,^35^. The PvRBP1 homologous domain was demonstrated to exhibit erythrocyte-binding activity in the 30 kDa PfRh4 ecto-domain^16^. Previously, full-length PvRBP1 was shown to exhibit reticulocyte-binding activity; however, the exact binding domain and its receptor on the reticulocyte surface are still unknown^8^. In this study, the binding domains of the PvRBP1a and PvRBP1b from PfRh4 binding domain homologue^16^ were confirmed and shown to possess reticulocyte-binding activity via binding domains that were not characterized in the previous study^8^. Although the sequence similarity to the PvRBP1a- and PfRh4-binding domains was relatively low (33.4%), the characterization of this homologous domain is important to clarify the erythrocyte binding activity of the PvRBP1 protein.

A previous study reported that native PvRBP1 was localized at the apical pole of the merozoite^8^. This study revealed the localization of PvRBP1a and PvRBP1b, which were expressed at the microneme similar to the *P. knowlesi* reticulocyte-binding proteins (PKH_146970 and PKH146980)^36^ but differed from other RBL family members, such as PfRh and PY235, which localized in the rhoptry^16^. Our findings suggest that PvRBP1 may have functional activity similar to that of the microneme protein PvDBP, which plays a role in junction formation during merozoite invasion of host cells. Another possibility is that PvRBP1 may participate in the cascade of events involved in invasion through specific interactions with a receptor on the reticulocyte surface during the release of microneme proteins.

The fragment representing PvRBP1 (435–777 aa.) contains the most polymorphic region of the protein, suggesting that it may be a target of functional antibodies^37^,^38^,^39^. In the present study, the PfRh4 homologue fragments PvRBP1a-34 (352–599 aa.) and PvRBP1b-32 (340–587 aa.) partially overlapped the PvRBP1 polymorphic region. PvRBP1a-34 and PvRBP1b-32 were observed to have low antigenicity with the vivax patient serum samples. Our results also confirmed the results from our previous study and another report^39^,^40^,^41^. PvRBP1b had relatively low antigenicity among the PvRBP family proteins compared with the high antigenicity of PvRBP2c^40^,^41^. The PvDBP-RII domain contains polymorphic sequences that may affect antibody recognition for protection against parasite invasion^42^,^43^. These results are consistent with the finding that *Plasmodium* polymorphic proteins are poorly immunogenic or elicit antibody responses that are short lived in the absence of frequent natural infections^38^. PvDBP-RII domain was also shown polymorphic sequences that may affect to antibodies recognition for the protection of parasite invasion because of the immune pressure^42^,^43^. Highly polymorphic domains of PvRBP1a and PvRBP1b may also possible to affect human antibodies recognition and not much effective for the protection of invasion similar properties as PvDBP-RII^39^. The study of anti-PvRBP1 and anti-PvDBP-RII antibodies seroprevalence in Brazil revealed predominantly cytophilic IgGs and significantly correlated with a number of past vivax malaria episodes, suggested that anti-PvRBP1 antibodies may have a long duration in repeat exposures^38^,^39^.

The most promising function of the PvRBPs is their reticulocyte-binding and selection activity^8^. In this study, fragments of a high-likelihood binding functional domain from PfRh4 were selected for the FACS-based binding assay with reticulocytes enriched by more than 70%. The PvRBP1a-34 and PvRBP1b-32 fragments exhibited specific binding activity with reticulocytes but not erythrocytes. A recent study of the PvRBP2a (160–460 aa.) fragment showed binding activities with both erythrocytes and reticulocytes^26^. These fragment structures shared the PfRh5 scaffold shape. These strong binding activities suggest that the PvRBP1a and PvRBP1b ligands have reticulocyte-selective activities that are dependent on an interaction with a specific, abundantly expressed reticulocyte receptor. PvDBP-RII showed stronger binding activity in reticulocytes than in erythrocytes. DARCs were more abundantly expressed on the reticulocytes and had an increased capacity to bind with specific receptors^44^. Although the identity of the PvRBP1a and PvRBP1b receptors is unclear, the present study clearly showed neuraminidase resistance in both proteins. These results suggest that PvRBP1a and PvRBP1b interact with a non-sialic acid receptor on the reticulocytes. PvRBP1b-32 showed both trypsin and chymotrypsin sensitivity, similar to PfRh4^16^. The enzyme-treated reticulocyte-binding assay showed significant inhibition of the binding activity in the presence of chymotrypsin; however, the binding activity was reduced by approximately 50% compared with the untreated samples. One possible explanation is that the abundant expression of the reticulocyte receptor overcame the enzyme reaction capacity. For example, the PvDBP-RII positive control also showed some binding following chymotrypsin treatment of the reticulocytes.

In conclusion, we proposed that PvRBP1a and PvRBP1b might play roles in specific reticulocyte binding through direct interactions between the recombinant proteins and the reticulocytes enriched from cord blood. These interactions contributed to the inhibition of immune serum samples, the binding specificity following enzyme treatment of the reticulocytes, the induction of relatively low IgG antibody responses in patients, and the localization of the proteins on the microneme of the apical organelles in schizont-stage parasites. Understanding the reticulocyte-binding antigen profile is important for elucidating the *P. vivax* invasion mechanisms. Future studies will determine the critical binding motifs of the PvRBP1a and PvRBP1b small molecules and evaluate the interaction receptor expressed on the reticulocytes during *P. vivax* invasion.

## Materials and Methods

### Human serum, parasite and cord blood samples

*P. vivax* patient samples were confirmed by microscopic examination (mean parasitemia, 0.12%). All samples were collected from the local hospital in Gangwon Province in malaria-endemic areas of the Republic of Korea (ROK) from 2008 to 2011. Healthy individual sera were collected from non-endemic areas. We used serum samples from 104 vivax patients and 72 healthy people for the humoral immune response analysis by protein array. Cord blood was collected in a 10 ml sodium heparin tube (BD Vacutainer^®^, Becton-Dickinson Co., NJ USA). All experiments were performed in accordance with relevant guidelines and regulations and all experimental protocols involving human samples approved by the Kangwon National University Hospital Ethical committee (IRB No. 2014-08-008-002). The written informed consent was obtained from all subjects.

### Expression and purification of the recombinant PvRBP1a-34 and PvRBP1b-32 proteins

Genomic DNA was extracted from a whole-blood sample from a parasite-infected patient. DNA was extracted from 200 μl of patient whole blood using the QIAamp DNA Blood Mini Kit (Qiagen, Hilden, Germany) following the manufacturer’s protocol and was used for amplification and subsequence cloning of the PvRBP1a and PvRBP1b genes. The primer sets were designed based on the *P. vivax* Sal-1 strain *pvrbp1a* (PlasmoDB ID: PVX_098585) and *pvrbp1b* (PVX_098582) sequences and contained a region homologous to the PfRh4-binding domain (PvRBP1a-34 and PvRBP1b-32): PvRBP1a forward 5′-AACGAACTAGGTATAGACATT-3′ and reverse 5′-ATTCAAACTCTATCTTCAGTTC-3′ (34 kDa fragment) and PvRBP1b forward 5′-GAAAGGGAGAATATAGACATTGCAG-3′ and reverse 5′-AATTCCCATGCACATTTTTCAA-3′ (32 kDa fragment). Each amplicon fragment was inserted into the pEU-E01-His-TEV-MCS vector (Cell Free Sciences, Matsuyama, Japan) containing a hexa-His tag at the N-terminus. Plasmid DNA fragments encoding PvRBP1a-34 (aa. position 352 to 599) and PvRBP1b-32 (aa. position 340 to 587) were generated and confirmed by sequencing analysis. These recombinant proteins were expressed using the wheat-germ cell-free expression system (Cell Free Sciences, Matsuyama, Japan) and purified using a Ni-affinity column (Qiagen) with an elution buffer containing 500 mM imidazole as previously described^45^,^46^. The PvDBP region II (RII) protein was expressed by *E. coli* and purified as previously described^47^. GST-His (glutathione S-transferase 6×His tag) protein expression was performed following the manufacturer’s protocol (GST Gene Fusion System, GE Healthcare Life Sciences, Uppsala, Sweden).

### Animal immune sera production

Female BALB/c mice (6 weeks old; Daehan Biolink Co., Eumsung, ROK) were used for the production of polyclonal antibodies against the recombinant PvRBP1a-34, PvRBP1b-32 and PvDBP-RII proteins. Groups of three mice were injected intraperitoneally with 20 μg of recombinant protein with Freund’s complete adjuvant (Sigma-Aldrich, St. Louis, MO, USA). Booster injections were given 3 and 5 weeks after the initial injection using the same amount of antigen with Freund’s incomplete adjuvant (Sigma-Aldrich), and mouse sera were collected 2 weeks after the final boost. To generate antibodies against PvRBP1a-34, PvRBP1b-32 and PvDBP-RII from rabbits, Japanese white rabbits were immunized subcutaneously with 250 μg of purified proteins with Freund’s complete adjuvant, followed by 250 μg with Freund’s incomplete adjuvant thereafter. All immunizations were administered three times at three-week intervals. The antisera were collected two weeks after the final boost. All animal experimental protocols were approved by the Institutional Animal Care and Use Committee of Kangwon National University, and the experiments were conducted according to the Ethical Guidelines for Animal Experiments of Kangwon National University (KIACUC-13-0001).

### SDS-PAGE and western blot analysis

Each recombinant protein (1 μg/well) was prepared in 2× reducing sample buffer, separated by 12% SDS-PAGE and then stained with Coomassie brilliant blue. For the western blot analysis, the proteins were transferred to PVDF membranes (Millipore, Bedford, MA, USA) by electrophoresis and incubated with blocking buffer (PBS containing 0.2% Tween 20 and 5% skim milk, PBS-T) overnight at 4 °C. The PVDF membrane containing recombinant proteins was incubated for 1.5 h at 37 °C with an anti-penta-His mouse antibody (1:2,000) (Qiagen) and animal immune serum (1:1,000) against each recombinant protein. After washing, the membrane was incubated with the goat anti-rabbit IRDye^®^ 680 (1:5,000) and goat anti-mouse IRDye^®^ 800 (1:10,000) antibodies (LI-COR Bioscience, Lincoln, NE, USA) in PBS-T for 1 h at 37 °C. To determine the reactivity of patient sera with the recombinant proteins, the recombinant proteins were probed with pooled sera from 10 patients at a 1:100 dilution in PBS-T and incubated with the goat anti-human IRDye^®^680 (1:10,000) antibody. Data were measured using the Odyssey infrared imaging system (LI-COR Bioscience) and analyzed with the Odyssey software (LI-COR Bioscience).

### Protein array

Three aminopropyl-coated slides were prepared as described previously^48^. Briefly, the slides were spotted and then each recombinant protein was applied to the spot at concentrations of 50 ng/μl for PvRBP1a-34 and 100 ng/μl for PvRBP1b-32. The slides were incubated for 2 h at 37 °C. After blocking, each spot was probed with 1 μl of patient or healthy serum (1:25 dilution in PBS-T) followed by incubation for 1 h at 37 °C. The arrays were visualized with 10 ng/μl of Alexa Fluor 546 goat anti-human IgG (Invitrogen, Carlsbad, CA, USA) in PBS-T for 1 h at 37 °C and scanned with a ScanArray Gx laser confocal scanner (PerkinElmer, Norwalk, CT, USA). The positive cut-off values of the negative control plus two standard deviations were used.

### Indirect immunofluorescence assay (IFA)

Mature schizont-stage parasites were purified from whole-blood samples from vivax malaria patients. The parasite antigen slides were prepared with ice-cold acetone for fixation and blocked with PBS containing 5% skim milk at 37 °C for 30 min. The following primary antibodies were used: rabbit anti-PvRBP1a-34 (1:50) and anti-PvRBP1b-32 (1:50) with anti-PvRAMA (1:200), anti-PvDBP-RII (1:100) and anti-PvMSP1-19 (1:200). The following secondary antibodies were used: Alexa 546-conjugated goat anti-mouse IgG or Alexa 488-conjugated goat anti-rabbit IgG (Invitrogen) at a 1:500 dilution. DAPI (4′,6′-diamidino-2-phenylindole, Invitrogen) was applied at a 1:1,000 dilution at 37 °C for 30 min to stain the nuclei. The slides were mounted with ProLong Gold antifade reagent (Invitrogen) and visualized under oil immersion using a Flowview^®^ FV1000 Laser Scanning Confocal imaging System (Olympus, Tokyo, Japan) equipped with a 60× oil objective. Images were captured using the FV10-ASW 3.0 viewer software and prepared for publication with Adobe Photoshop CS5 (Adobe Systems, San Jose, CA, USA). Each fluorescence graphic contained more than 500 pixels calculated by Image J (NIH, USA).

### Reticulocyte enrichment from cord blood

Reticulocytes were enriched from heparinized umbilical cord blood with a 19% Nycodenz solution (Axis-Shield, Oslo, Norway) in high-KCl buffer using gradient centrifugation^49^. Fresh cord blood was washed twice with incomplete RPMI 1640 medium, and white blood cells were removed using an NWF filter (Zhixing Bio Co. LTD., Bengbu, China). After centrifugation, packed cells were resuspended with a high-KCl buffer (pH 7.4) and then incubated at 4 °C for 3 h with rotation on a shaking rocker. Nycodenz solution (19%, 3 ml) was transferred into 15 ml tubes. Then, 5 ml of the RBC-high KCl buffer mixture was added on top of the Nycodenz solution layer and centrifuged at 3,000× g for 30 min without braking. The reticulocytes in the interface layer were harvested and washed three times with RPMI 1640 medium. The reticulocyte purity was calculated from thin blood smear samples with new methylene blue stain under light microscopy and thiazole orange (TO) staining of the enriched reticulocytes. A total of 200,000 events were acquired per sample using the FACS Accuri™ C6 Flow Cytometer (Becton-Dickinson Co., Mansfield, MA, USA).

### Enzyme treatment of RBCs

Reticulocyte-enriched samples were prepared with up to 50% hematocrit. The RBCs were washed with 500 μl of incomplete RPMI 1640 medium twice by centrifugation at 500× g for 3 min at 4 °C. The RBCs were incubated with neuraminidase (from *Vibrio cholera*, Sigma-Aldrich), trypsin (from bovine pancreas, Sigma-Aldrich) and chymotrypsin (from bovine pancreas, Sigma-Aldrich) at 37 °C on a rotator for 1 h. The trypsin and chymotrypsin-treated RBCs were incubated with a trypsin inhibitor (from the *Glycine max* soybean, Sigma) at 37 °C for 10 min. The RBC samples were washed twice with 10 ml of incomplete RPMI 1640 medium. The packed cells were prepared at a concentration of 1 × 10^6 ^cell/ml and used for flow cytometry analysis.

### Reticulocyte-binding assay by flow cytometry

The reticulocyte-enriched samples were used for the flow cytometry-based direct-binding assay. Briefly, 1 × 10^6^ reticulocytes/ml or the same concentration of reticulocytes treated with each enzyme was incubated for 4 h at 25 °C with 0 to 20 μg of hexa-His-tagged recombinant PvRBP1a-34 or PvRBP1b-32 protein. PvDBP-RII and GST-His were used as the positive and negative controls for the reticulocyte binding assay, respectively. The samples were washed twice with 200 μl of PBS containing 1% BSA (PBS-BSA) and incubated with a mouse anti-penta-His Alexa Fluor 647-conjugated monoclonal antibody (Qiagen) for 1 h at 4 °C in the dark. The samples were washed three times with PBS-BSA and incubated with the Thiazole Orange (TO) Retic-COUNT reagent (Becton-Dickinson Co., San Jose, CA, USA) for 30 min at 25 °C. For the fluorescent detection of each RBC, a total of 100,000 events were acquired per sample using the FACS Accuri™ C6 Flow Cytometer (BD). The flow cytometric results were analyzed using FlowJo 7.6 (Treestar, Ashland, OR, USA). Unstained cells and cells singly stained with TO were used to separate the normocytes and reticulocytes, respectively.

### Statistical analysis

The data were analyzed using GraphPad Prism (GraphPad Software, San Diego, CA, USA), SigmaPlot (Systat Software Inc., San Jose, CA, USA), and Microsoft Excel 2013 (Microsoft, Redmond, WA, USA). Two-dimensional scatter data were obtained as a simple scatter with a regression graph of *x, y* axis pairs^50^. For the protein array, Student’s *t*-test was used to compare the experimentally measured values of each group. Differences of *p* < 0.05 were considered significant.

## Additional Information

**How to cite this article**: Han, J.-H. *et al.* Identification of a reticulocyte-specific binding domain of *Plasmodium vivax* reticulocyte-binding protein 1 that is homologous to the PfRh4 erythrocyte-binding domain. *Sci. Rep.* **6**, 26993; doi: 10.1038/srep26993 (2016).

## Supplementary Material

Supplementary Information

## Figures and Tables

**Table 1:** Prevalence, 95% confidence intervals, and mean fluorescence intensity of IgG responses to each fragment of *P. vivax* DBP-RII, RBP1a-34 and RBP1b-32 in human patients and healthy individual serum samples.

Antigen	No. of patient samples			95% CI^b^	MFI^c^	No. of healthy samples			95% CI	MFI	*p* value^e^
	Positive	Negative	Total (%)^a^			Positive	Negative	Total (%)^d^			
PvDBP-RII	41	31	72 (56.9)	45.4–67.7	5968.4	1	23	24 (95.8)	79.8–99.3	2769.5	*p* < 0.0001
PvRBP1a-34	35	69	104 (33.7)	25.3–43.2	5513.7	3	69	72 (95.8)	88.5–98.6	3593.4	*p* < 0.0001
PvRBP1b-32	41	63	104 (39.4)	30.6–49.0	5452.2	3	69	72 (95.8)	88.5–98.6	2939.6	*p* < 0.0001

^a^Sensitivity: percentage of positive in patient samples.

^b^CI: confidence interval.

^c^MFI: mean fluorescence intensity.

^d^Specificity: percentage of negative in healthy samples.

^e^Differences in the total IgG prevalence for each antigen between vivax patients and healthy individuals were calculated with Student’s *t*-test. A *p* value of <0.05 is considered statistically significant.
