# Supplementary material for: Identification of a reticulocyte-specific binding domain of Plasmodium vivax reticulocyte-binding protein 1 that is homologous to the PfRh4 erythrocyte-binding domain
Source: Sci Rep. 2016 May 31;6:26993. doi: 10.1038/srep26993 (PMC4886630; doi:10.1038/srep26993)
Supplement: Supplementary Information [file srep26993-s1.pdf]

Supplementary Information

Identification of reticulocyte specific binding domain of *Plasmodium vivax* reticulocyte binding protein 1 (PvRBP1) homologus to PfRh4 erythrocyte binding domain

Jin-Hee Han[1], Seong-Kyun Lee[1], Bo Wang[1,2], Fauzi Muh[1], Myat Htut Nyunt[1,3], Sunghun Na[4], Kwon-Soo Ha[5], Seok-Ho Hong[6], Won Sun Park[7], Jetsumon Sattabongkot[8], Takafumi Tsuboi[9], Eun-Taek Han[1*]

Supplementary Fig. 1

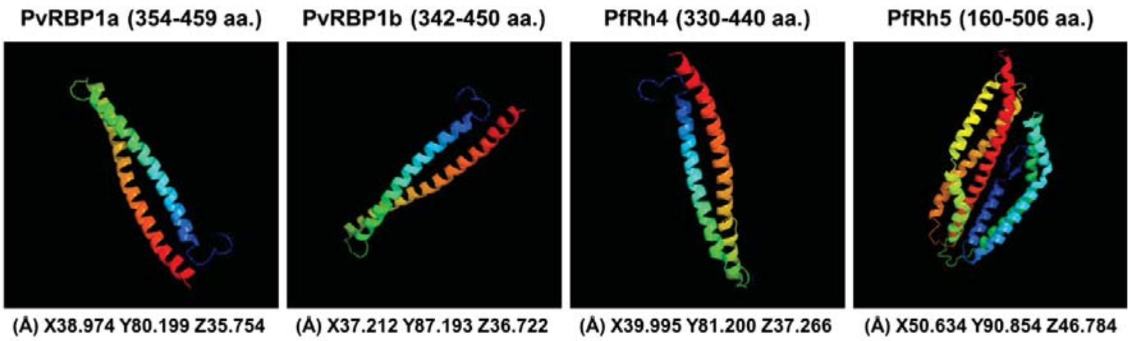

Fig. S1 Reticulocyte binding like proteins in *Plasmodium* spp. 3D structure prediction by Phyre2 web portal (http://www.sbg.bio.ic.ac.uk/phyre2/html/help.cgi?id=help/faq). The template identified PfRh5 (c4u1gA) which bound to the monoclonal antibody. PvRBP1a, PvRBP1b and PfRh4 domains showed more than 99.5% confidence with PfRh5.
